# A Study of the Mechanical Properties of Gas Hydrate-Bearing Sediments Under Membrane Boundary Conditions Using the Discrete Element Method

**DOI:** 10.3390/ma19214479

**Published:** 2026-10-24

**Authors:** Xiuxing Zhu, Jie Cui, Lei Liu, Bing Han, Jiahao Li, Bo Zhou

**Affiliations:** College of Pipeline and Civil Engineering, China University of Petroleum (East China), Qingdao 266580, China

**Keywords:** gas hydrate, membrane boundary condition, discrete element method, biaxial test, macroscopic mechanical property, mesoscopic mechanical property

## Abstract

Understanding the mechanical behavior of gas hydrate-bearing sediments (GHBSs) is critical for safe hydrate extraction. This study employs the discrete element method (DEM) in PFC2D to investigate the macro- and meso-mechanical properties of GHBSs. A novel layered staining technique is proposed to enhance deformation visualization in membrane-bound GHBS samples. Biaxial simulations were conducted on samples with varying hydrate saturations. Results demonstrate that the deviatoric stress–axial strain curves exhibit strength enhancement with increasing hydrate saturation, while volumetric strain–axial strain curves reveal distinct contraction–dilation behavior. Shear band formation observed during failure highlights the shear-dominated damage mechanism. At the mesoscale, key parameters—coordination number, deviatoric fabric, contact force, and particle rotation—were systematically analyzed. The coordination number initially increases and subsequently decreases with axial strain, correlating with the contraction–dilation transition. Similarly, deviatoric fabric and contact force evolution align with the hardening–softening characteristics of GHBSs. Notably, intensified particle rotation within shear bands underscores its pivotal role in shear localization. These findings bridge mesoscopic mechanisms to macroscopic behavior, providing insights for optimizing hydrate extraction strategies. It should be noted that the simulations are two-dimensional plane–strain idealizations and adopt a single hydrate occurrence pattern; the quantitative results are therefore conditioned by these assumptions, and their extension to other occurrence patterns and to three dimensions is left for future work.

## 1. Introduction

Gas hydrate-bearing sediments (GHBSs) are compounds that form when water and natural gas combine at high pressures and low temperatures. GHBSs exhibit exceptional energy density, as 1 cubic meter of GHBS can store 164 cubic meters of methane-containing energy [1]. Due to their high energy density and substantial reserves [2], GHBSs are increasingly recognized as a highly promising and viable energy resource for extraction. However, the extraction of natural gas hydrates poses challenges, as they are inherently unstable and can impact the geological environment in which they reside. This can result in hydrate decomposition and give rise to various geological hazards [3,4]. Consequently, an examination of the mechanical properties of GHBSs is crucial to ensure their secure exploitation.

In recent decades, researchers have conducted numerous experimental investigations into the mechanical properties of gas hydrate-bearing sediments [5,6,7,8,9,10]. These studies have primarily utilized triaxial or biaxial compression experiments to explore the factors impacting the mechanical properties of gas hydrate-bearing sediments (GHBSs). It has been observed that the strength of GHBS is closely influenced by the distribution, saturation, and experimental conditions of hydrates. The presence of hydrates can enhance the stiffness, shear dilation, and peak stress of GHBS. Moreover, laboratory preparation of GHBS is difficult, and it is highly susceptible to decomposition. Discrete element method (DEM) numerical simulations are gradually becoming a popular method of studying this problem.

The discrete element method (DEM) has been widely employed by numerous researchers to investigate the mechanical properties of GHBS in recent years [11,12,13,14,15]. DEM simulations have provided valuable insights into the behavior of GHBS, yielding results in good agreement with experimental findings. For instance, Brugada et al. [11] successfully simulated the triaxial compression test of GHBS using the DEM and investigated the effects of hydrate saturation and confining pressure on reservoir strength and stiffness. Similarly, Jiang et al. [13] developed a DEM numerical simulation model for GHBS and investigated its deformation mechanism. Wang et al. [14] focused on the evolution of GHBS microstructure during shear by conducting a series of drainage cycle biaxial compression tests via the DEM method. Furthermore, Zhou et al. [15] examined the shear behavior of GHBS under undrained conditions using the DEM and obtained significant insights into its macroscopic and mesoscopic mechanical responses.

Indeed, in the aforementioned studies utilizing the discrete element method (DEM) to investigate the mechanical properties of gas hydrate-bearing sediments (GHBS), the lateral boundaries of the samples were assumed to be rigid [16], whereas GHBS specimens in physical tests are surrounded by a soft rubber membrane that maintains the target confining pressure and allows the free deformation of the sample and the development of shear bands [17]. Motivated by this gap, the present study investigates the macro- and meso-mechanical properties of GHBS under a membrane boundary condition using the DEM in PFC2D. The primary objective is to reproduce the macroscopic response of GHBS in biaxial compression, including the deviatoric stress–strain behaviour, the volumetric response, and the formation of shear bands, under a flexible boundary consistent with physical tests, and to calibrate the numerical model against the experiments of Masui et al. [7]. The secondary objective is to explain the macroscopic behaviour on the mesoscale by examining the evolutions of the coordination number, the deviatoric fabric, the contact force distribution, and particle rotation.

## 2. Discrete Element Method (DEM)

In this paper, DEM (discrete element method) software is employed to prepare numerical samples of gas hydrate-bearing sediments (GHBSs). The preparation process consists of three stages: preparation of the initial sample, application of the contact model, and layered staining.

### 2.1. Preparation of the Initial Sample

Figure 1 illustrates the characteristics of a numerical sample of gas hydrate-bearing sediments (GHBSs) with a hydrate saturation of 40.0%. The sample is represented as circular disks representing hydrate and soil particles. The sample is contained within a rectangular box measuring 24.00 mm in height and 12.00 mm in width. Figure 1b depicts the arrangement of hydrate and soil particles within the sample. The soil particles are represented by brown disks, while the hydrate particles are depicted as red and blue disks. It is worth noting that the hydrate particles initially generated as floaters, represented by red disks, if the distance between them and other particles is less than 2. This distinction is made to identify which hydrate particles are not in contact with other particles. When floaters are found, they will come into contact with other particles to make them non-floaters and are marked as blue disks, as shown in Figure 1c. This process ensures that all hydrate particles are in contact with other particles within the GHBS sample, facilitating their application to the contact model employed in the subsequent steps.

In Figure 2, the particle size distribution curves of soil particles are presented. The diameter of the soil particles varies between 0.14 and 0.40 mm. This range is determined based on the sand grading curve of Toorak sand, a reference material commonly used in geotechnical engineering studies. It is important to note that Figure 2 specifically represents the particle size distribution of the soil particles and does not include the hydrate particles. The diameter of the hydrate particles, however, is specified separately as 0.06 mm. By providing information on particle size distributions, the authors offer valuable insights into the composition and characteristics of the GHBS sample, which play a significant role in determining the mechanical behavior and response of the sediment under study.

Figure 3 displays the numerical samples of gas hydrate-bearing sediments (GHBSs) prepared with hydrate saturations of 26.0%, 40.0%, and 55.0%. We selected these particular hydrate saturations in order to enable a comparison with experimental results obtained by Masui et al. [7]. In their study, GHBS samples were prepared with hydrate saturations of 25.7%, 40.7%, and 55.1%, respectively. It is noted that as the hydrate saturation decreases, there is a greater presence of floaters. Floaters can pose challenges when applying the contact model; they are therefore screened out and moved into contact with neighbouring particles, following the procedure described for Figure 1c.

### 2.2. Application of the Contact Model

What type of contact should be imposed between different particles is an important issue in DEM simulation. In this paper, two contact models are used: the linear model [18] and the linear parallel bond model [19]. Figure 4 provides a depiction of the mechanical components comprising the utilized contact model.

As shown in Figure 4b, the linear model provides both linear and dashpot components. The linear component provides linear elastic frictional behavior but cannot sustain tension, while the dashpot component provides viscous behavior. The linear component consists of linear springs with constant normal and shear stiffnesses (*k_n_* and *k_s_*), a sliding element with friction coefficient *μ*, and a pressure bearing element for identifying the normal relative displacement *g_s_* between the particles. The damping component is composed of damping element with normal and shear critical-damping ratios, *β*_n_ and *β*_s_. Those components act over a small area and primarily transmit forces.

As shown in Figure 4c, the linear parallel bond model consists of a linear component and a parallel bond component. The parallel bond component acts in parallel with the linear component, and parallel bonds can transmit both force and moment. The linear component is the same as the linear contact model. The parallel bond component is composed of linear springs with constant normal and shear stiffnesses (k_n_* and k_s_*), a tensile element with tensile strength σ_c_*, a shear resistant element with cohesion c*, and friction angle ϕ*. When the bond breaks, the linear parallel bond model is equivalent to the linear model. The parallel bond part acts on a circular plane at the contact point.

Gas hydrates exhibit a cementation effect between particles. In order to capture the unique bonding behavior and interactions specific to gas hydrates, the linear parallel bond model is specifically applied to the hydrate–hydrate and hydrate–soil contacts in the simulation. The linear model, on the other hand, is applied to the sand–sand contacts.

To simulate the initial state of GHBS in the stratum, a confining pressure of 1 MPa is applied to the samples. Since the force cannot be applied directly to the particles and the surrounding wall in the PFC^2D^ 5.0 software, it is necessary to use the principle of servo control in the software to apply the velocity to the wall so as to apply the confining pressure.

Before the mechanical responses are interpreted, the hydrate occurrence pattern adopted in this study should be clarified. According to the generation procedure described in Section 2.1 and the contact model assignment described in Section 2.2, the sample used here does not correspond to a pure floating (pore-floating) pattern, in which hydrate merely occupies the pore space without bonding to the surrounding grains. Hydrate particles are first generated as floaters inside the pores and are then moved into contact with the adjacent soil or hydrate particles; subsequently, linear parallel bonds with a tensile strength of 50 MPa and a cohesion of 40 MPa are installed at hydrate–hydrate and hydrate–soil contacts, whereas soil–soil contacts remain frictional. The hydrate therefore affects the specimen response in two ways: it fills the pores and increases the local packing density and the number of contacts, and it cements the contacts and transmits force and moment, thereby restraining particle sliding and rotation. This combination of pore filling and contact cementation is referred to as the mixed occurrence pattern in the following discussion.

The adoption of this mixed occurrence pattern is justified by two considerations. First, it is consistent with the hydrate saturations of the calibration experiments (25.7%, 40.7%, and 55.1%) reported by Masui et al. [7], so that the microscopic parameters in Table 1 are calibrated against a comparable hydrate morphology rather than an arbitrary one. Second, pore filling accompanied by inter-particle cementation is a representative morphology of hydrate in sand-dominated sediments, in which hydrate occupies the pore space and bonds the adjacent grains. It should nevertheless be emphasized that hydrate may also occur in other patterns, for example grain-coating or load-bearing patterns, in which hydrate envelopes the grains or participates directly in the force chains. For these patterns, the absolute values of the macroscopic strength, the elastic modulus, the dilation magnitude and the characteristic strains reported in Section 3 and Section 4 are expected to change, because both the bonded contact area and the stiffness of the hydrate phase differ. The qualitative trends identified in this study—namely, the enhancement of strength and stiffness with increasing hydrate saturation, the contraction–dilation transition, the hardening–softening behaviour, and the localization of deformation into shear bands—are largely controlled by the evolution of the granular skeleton and are therefore expected to persist, although their quantitative expression may differ. A systematic comparison between occurrence patterns is beyond the scope of the present study and is left for future work.

### 2.3. Layered Staining of Sample

A layered staining sample method is proposed to observe the subsequent shear deformation of the sample. Boundary conditions play an important role in capturing the correct mechanical behavior of GHBS. Rigid walls are usually used as boundary conditions for discrete element simulation. But the GHBS sample is always surrounded by a rubber membrane in a physical biaxial test, which means that the boundary of the GHBS is a flexible boundary. Flexible boundaries can capture the deformation of the sample and the development of shear band, which will be presented in the subsequent sections. Therefore, the flexible boundary conditions of the sample need to be established in the PFC^2D^ software.

It should be noted that the layered staining shown in Figure 5 is a purely optical measure for deformation visualization and does not alter the mechanical response of the sample. The particles belonging to the green and brown domains share the same size distribution, density and contact parameters, and the contacts across the boundaries between adjacent stained domains are treated in exactly the same way as those inside a domain. No mechanical heterogeneity, weak plane, or interface is therefore introduced by the staining: the green and brown squares only provide a material coordinate grid against which the distortion of the sample, and hence the development of the shear band, can be identified by eye.

Figure 5 shows a layered stained sample under the membrane boundary condition. Green and brown particles are soil particles. Blue particles are hydrate particles. The sample is divided into a number of green and brown squares consisting of particles. Two rows of yellow membrane particles are used to represent the flexible boundary condition. The yellow circular particles are regarded as continuous membranes. The diameter of the membrane particle is 0.06 mm, which is consistent with the diameter of the hydrate particles. The elastic modulus of the membrane particles is set to 8 MPa, which is similar to the elastic modulus of rubber. The tensile strength and shear strengths are set to 1.0 × 10^294^ MPa. This value is tens of orders of magnitude larger than any stress arising in the simulation, so that the bonds of the membrane particles are effectively unbreakable and the membrane behaves as a pure flexible boundary throughout loading. Since the bond strength is far above the stress level of the problem, the exact value of the exponent is immaterial for the results; the membrane particles simply act as elastic connectors between the confining pressure and the sample. The top and bottom caps consist of rigid walls with an effective modulus of 1200.0 MPa.

Figure 5c shows the external force generated by the confining pressure on the membrane particles. The diameter of the membrane particle is the length of the contact, *D*. The confining pressure is defined as *σ*_3_, and the force on the whole membrane by confining pressure is defined as *F*. Then, *F* can be calculated by

(1) $$F=\frac{1}{2}\sigma_{3}D$$

The force is applied to the membrane particles in the PFC^2D^ software according to Equation (1). The walls above and below also need to be loaded according to the servo principle, so as to achieve the purpose of loading the sample by applying 1 MPa of confining pressure. After that, the biaxial simulation test under the membrane boundary condition of GHBS is completed.

## 3. Macroscopic Mechanical Properties

### 3.1. Stress and Strain Relationship

Biaxial tests of samples with different hydrate saturations are simulated using the discrete element method through PFC^2D^ software. The curves of deviatoric stress versus axial strain with different hydrate saturations are obtained. In the two-dimensional case, the deviatoric stress *q* is defined as

*q* = *σ*_1_ − *σ*_3_ (2)

where *σ*_1_ is the axial stress, and *σ*_3_ is the confining pressure on the side walls.

The axial strain is defined by

(3) $$\varepsilon_{1}=\frac{l-l_{0}}{l_{0}}$$

where *l*_0_ is the initial height of the sample, and *l* is the height during shearing.

In order to calibrate the microscopic parameters, the simulation results will be compared with experimental results from Masui et al. [7]. Since determining these microcosmic parameters is challenging, a series of trial calculations and calibrations are conducted through trial and error. The parameters utilized in the simulations are presented in Table 1.

Figure 6 depicts the numerical test results as lines and the physical test results as discrete points. The numerical simulated results align well with the experimentally obtained results. The symbol ‘Sh’ is utilized to represent the hydrate saturation of GHBS, which is subsequently used throughout. During the initial loading stage, the deviatoric stress exhibits an approximately linear increase with the axial strain. However, as the deviatoric stress reaches its maximum value, a strain softening phenomenon emerges. The macroscopic elastic modulus and peak strength of GHBS demonstrate an increase in correlation with the increase in Sh. Notably, the presence of gas hydrate enhances the strength and stiffness of GHBS.

### 3.2. Volumetric Strain Response

The volumetric strain is defined by

(4) $$\varepsilon_{v}=\frac{V-V_{0}}{V_{0}}$$

where *V*_0_ is the fixed initial volume of the sample, which is known from the sample geometry before loading, and *V* is the current volume of the sample, which varies continuously as loading proceeds. *V*_0_ is a constant and is therefore known. In contrast, the current volume changes as the loading proceeds, so measuring *V* at every loading step is a challenge.

The triangle method, which is commonly used in CAD and other calculation software, is used here to calculate *V*. Since this paper simulates the 2D case, the change in volume is the change in area.

Figure 7a shows that the sample is divided into four parts to calculate the volume after deformation. S_top_ and S_bottom_ always remain triangular during the deformation of the sample, so the area can be calculated directly. But S_left_ and S_right_ are irregular polygons during the deformation of the sample.

Figure 7b indicates that the centers of two adjacent particles are connected to the center of the sample to form many triangles. If the vertices of the triangle are arranged in a counterclockwise direction, as in the blue triangle depicted in Figure 7b, the corresponding area should be subtracted during the overall area calculation. On the contrary, if the vertices of the triangle are aligned in a clockwise direction, as in the red triangle illustrated in Figure 7b, the area should be added to the total area calculation. This method is used to program the PFC2D software to calculate the volumetric strain of the deformed GHBS sample.

Figure 8 displays the volumetric strain versus axial strain curves. The samples demonstrate a pattern of shear shrinkage initially, followed by shear dilation. At the beginning, a slight shear shrinkage is observed, which is subsequently followed by significant shear dilation. It is worth noting that the degree of GHBS dilation increases with the rising hydrate saturation.

### 3.3. Shear Band

During the occurrence of damage in granular materials, strain tends to concentrate within a localized region, leading to the formation of a shear band. The samples are stained in layers to facilitate the observation of the evolution of the shear band.

Figure 9 shows the evolution of the shear band of the sample. With the increase in axial strain, the sample starts to expand and deform gradually. When the axial strain increases by 2%, a shear band starts to appear. The higher the axial strain is, the more pronounced the shear band is. When the axial strain increases by 8%, it is clear that the two shear bands begin to form an X shape. When the axial strain reaches 12%, the shear band of the X shape is fully formed. The formation of shear bands can also be seen indirectly through the deformation of the initially set green and brown granular squares.

The origin of the X-shaped pattern deserves a comment. Since the stained domains are arranged in a regular and periodically repeated manner (Figure 5), we may question whether the diagonal directions of the domain arrangement bias the orientation of the shear bands. Several observations indicate that this is not the case. The layered staining only provides a visualization grid, and, as stated in Section 2.3, it changes neither the contact parameters nor the local packing, so that no plane of weakness exists along the diagonals of the staining pattern. The same localization is reproduced by mesoscopic quantities that are completely independent of the staining: the principal direction of the normal contact force distribution remains between 83 degrees and 86 degrees with respect to the horizontal axis (Table 2). The X-shaped shear band is therefore a consequence of the kinematics of the granular assembly under biaxial compression rather than an artifact of the domain arrangement used for staining.

## 4. Mesoscopic Mechanical Properties

### 4.1. Coordination Number

As loading proceeds, the particles inside the sample move and rotate, which causes the particles to rearrange. The number of contacts between particles changes. The average coordination number *Z* is used to characterize the evolution of the number of contacts during loading, which is defined by the following equation:

(5) $$Z=\frac{2C}{N}$$

where *C* is the number of contacts, and *N* is the number of particles.

In Equation (5), *Z* is a parameter used to indicate the degree of contact. The larger its value, the more adequate the contact is and the more stable the particle system is. However, the discrete element simulation results show that some particles do not form contact with the surrounding particles or have only one contact. These particles do not contribute to the stability of the particle system. Hence, a mechanical average coordination number is defined [20]:

(6) $$Z_{m}=\frac{2C-N_{1}}{N-N_{0}-N_{1}}$$

where *N*_0_ and *N*_1_ are the number of particles with no contact or only one contact, respectively. This index indicates the average number of contact points of all skeleton particles, which can reflect the denseness of the granular skeleton inside the sample.

Figure 10 shows the curves of the evolution of coordination number and mechanical coordination number inside the sample with axial strain at different hydrate saturations. The coordination number and mechanical coordination number increase and then decrease with the increase in the axial strain, which is consistent with the contracting–dilatancy properties. As the axial strain increases, the sample begins to shrink and the pores inside the sample are compacted, making the contact between the particles more adequate and causing the coordination number and mechanical coordination number to increase. After that, the sample starts to dilate, the particles gradually start to separate from the surrounding particles, and the coordination number and mechanical coordination number decrease. Both the coordination number and the mechanical coordination number tend to stabilize in the final stage, indicating that the contact between particles will also stabilize. The greater the saturation of the hydrate is, the more adequate the particle contact is and the larger the coordination number and mechanical coordination number are.

### 4.2. Deviatoric Fabric

The gas hydrate-bearing sediments consisting of hydrate particles and soil particles are essentially anisotropic granular materials. The constitutive tensor is often used to describe the anisotropy of granular materials [21]. The contact normal is highly sensitive to stress changes and adjusts its spatial distribution as the external load changes; the contact normal is used to define the fabric tensor [22].

As shown in Figure 11, the contact normal direction is in the same direction as the line connecting the centers of the two particles, and the unit vector *n* = (*n_x_*,*n_y_*) along this direction is defined as the unit contact normal. The fabric tensor is defined by the following equation [23]:

(7) $$\Phi_{\mathrm{ij}}=\frac{1}{N}\sum_{k=1}^{N}n_{i}^{k}n_{j}^{k}$$

where *N* is the total number of contacts in the sample, and $k_{n}^{i}$ (*i* = *x*, *y*) is the value of the k-th contact normal unit vector coordinate value. The fabric tensor represents the quantitative statistics of the spatial distribution of all contact normal.

The fabric tensor defined by Equation (7) has three eigenvectors and three eigenvalues. The eigenvectors represent the dominant directions of fabric anisotropy, while the eigenvalues indicate the magnitude of the bias in each of these directions. The eigenvalues are defined as Φ_1_, Φ_2_ and Φ_3_ and satisfy Φ_1_ > Φ_2_ > Φ_3_. The difference between the values of Φ_1_, Φ_2_ and Φ_3_ is a measure of the magnitude of fabric anisotropy. Thus, the deviator tensor Φ_d_ is defined as follows [24]:

(8) $$\Phi_{d}=\frac{1}{\sqrt{2}}{\left[{\left(\Phi_{1}-\Phi_{2}\right)}^{2}+{\left(\Phi_{2}-\Phi_{3}\right)}^{2}+{\left(\Phi_{1}-\Phi_{3}\right)}^{2}\right]}^{0.5}$$

Figure 12 shows the curves of deviatoric fabric versus axial strain of the GHBS with different hydrate saturations. The samples have initial anisotropy due to the pre-pressing at the beginning, so the sample has an initial deviatoric fabric value. The deviatoric fabric increases and then decreases with the increase in axial strain, which is consistent with the hardening and softening properties. This is because the anisotropy of the material will change with the external load. When the stress increases, the movement and rotation of the particles will become more pronounced, and the material will become more disorganized inside; the anisotropy of the material will also increase. The greater the hydrate saturation of GHBS, the greater the value of the deviatoric fabric, and the greater the anisotropy of GHBS. This phenomenon indicates that gas hydrate dominates the anisotropy of GHBS.

### 4.3. Contact Force

Taking GHBS with 40.0% hydrate saturation as an example, the contact forces at different axial strains are statistically grouped to visualize the evolution of contact force distribution in GHBS. The distribution of normal contact force and tangential contact force are plotted, as shown in Figure 13 and Figure 14. In order to quantitatively describe the variation of anisotropy within the sample, the Fourier distribution function [25] is used to fit the inter-particle contact force statistics:

*f_n_*(*θ*) = *f*_0_[1 + *a_n_*cos2(*θ* − *θ_n_*)] (9)

*f_t_*(*θ*) = −*f*_0_*a_t_*sin2(*θ* − *θ_t_*) (10)

where *f_n_*(*θ*) and *f_t_*(*θ*) are the normal and tangential contact force distribution functions between particles, respectively. *f*_0_ is the average normal contact force over all contacts. *a_n_* and *a_t_* are the Fourier coefficients indicating the degree of anisotropy of normal and tangential contact force distributions, respectively. *θ_n_* and *θ_t_* are the principal directions of normal and tangential contact force distribution, respectively. The negative sign in Equation (10) represents the positive tangential force tending to rotate the particle counter-clockwise. The statistical distribution of inter-particle contact forces under different axial strains is fitted according to Equations (9) and (10). The fitted parameters are shown in Table 2.

As shown in Figure 13 and Table 2, the evolution of the normal contact force distribution between the particles inside the sample is consistent with the hardening and softening properties of GHBS. In the initial stage, the sample is in a solidified state, which is isotropic, so the anisotropy parameter *a_n_* is almost zero and the contact force distribution is approximately circular. With the increase in axial strain, anisotropy appears inside the sample, and the normal contact force distribution pattern gradually changes to peanut-shaped [26]. The larger average normal contact force is concentrated in the direction of about 90°, i.e., the axial loading direction. Before the deviatoric stress reaches its peak, i.e., the axial strain is less than 2%; *f*_0_ and *a_n_* increase with the increase in axial strain. They decrease with the increase in axial strain when the axial strain is more than 2%. This trend is consistent with the evolution of the curve of deviatoric stress versus axial strain.

Similarly, from Figure 14 and Table 2, it can be concluded that the tangential contact force distribution also has the same evolutionary pattern. In the initial stage, the tangential contact force between particles is almost zero. With the increase in axial strain, the sample is in shear mode, and the tangential contact force distribution between the particles appears petal-like. *f*_0_ and *a_t_* increase with the increase in the axial strain when the axial strain is less than 2%. They decrease with the increase in the axial strain when the axial strain is more than 2%.

### 4.4. Particle Rotation

The rotation of material particles has an important influence on the process of shear band emergence and development [27]. Taking the sample with 40% hydrate saturation as an example, the rotation angle diagram of the particles is derived from the PFC software.

As shown in Figure 15, the rotation of the particles is inextricably linked to the formation of shear bands. With the increase in axial strain, the particles on the shear band start to rotate. The greater the axial strain is, the more violent the rotation is. Those particles that are not on the shear band barely rotate. The rotation direction of the particles on both sides of the X-shaped shear band is opposite. The greater the tendency to destroy, the greater the angle of rotation of the particle is. An interesting phenomenon is observed: the membrane particles on the boundary rotate very vigorously, and the rotation of two adjacent particles is in opposite directions.

## 5. Conclusions

This study investigates the shear behavior of gas hydrate-bearing sediments (GHBSs) through discrete element method (DEM) simulations of biaxial tests under membrane boundary conditions. A layered staining method is introduced to enhance visualization of shear band evolution during deformation. Our key findings are summarized as follows.

(1) DEM simulations effectively predict GHBS mechanical properties, demonstrating that increased hydrate saturation enhances both the stiffness and peak strength of the specimens.
(2) Biaxial shear processes exhibit sequential contraction–dilation behavior, with shear dilation becoming more pronounced at higher hydrate saturations.
(3) During the loading process, the coordination number and mechanical coordination number of GHBS first increase and then decrease, which is consistent with the contracting–dilatancy phenomenon.
(4) The distributions of the normal and tangential contact forces are peanut-shaped and petal-shaped, respectively, and their values first increase and then decrease, in accordance with their hardening and softening properties.
(5) With the increase in axial strain, the sample gradually forms an X-shaped shear band, and the particle rotation in the shear band is more violent than in other parts, which indicates that particle rotation plays an important role in forming the shear band.
(6) The quantitative strength and stiffness values reported above are conditioned by the mixed occurrence pattern (pore filling combined with contact cementation) adopted in this study and by the calibrated bond strength; the qualitative trends summarized in items (1)–(5) are expected to persist for other hydrate occurrence patterns, but a systematic comparison between occurrence patterns remains to be carried out.

Several limitations of this study follow from the methods employed and should be kept in mind when the results are used. The simulations are two-dimensional plane–strain idealizations with circular particles, so the true three-dimensional fabric, the particle shape, and the intermediate principal stress are not represented. The hydrate is modelled as bonded disks with a single occurrence pattern (pore filling combined with contact cementation), and hydrate decomposition as well as temperature and pore pressure effects are not considered. The quantitative values of strength, stiffness, and dilatancy reported above should therefore be interpreted within these assumptions. Future research will extend the model to three dimensions, compare different hydrate occurrence patterns (e.g., grain-coating and load-bearing patterns), and incorporate thermo-hydro-mechanical coupling to describe hydrate dissociation during extraction.

## Figures and Tables

**Figure 1:**
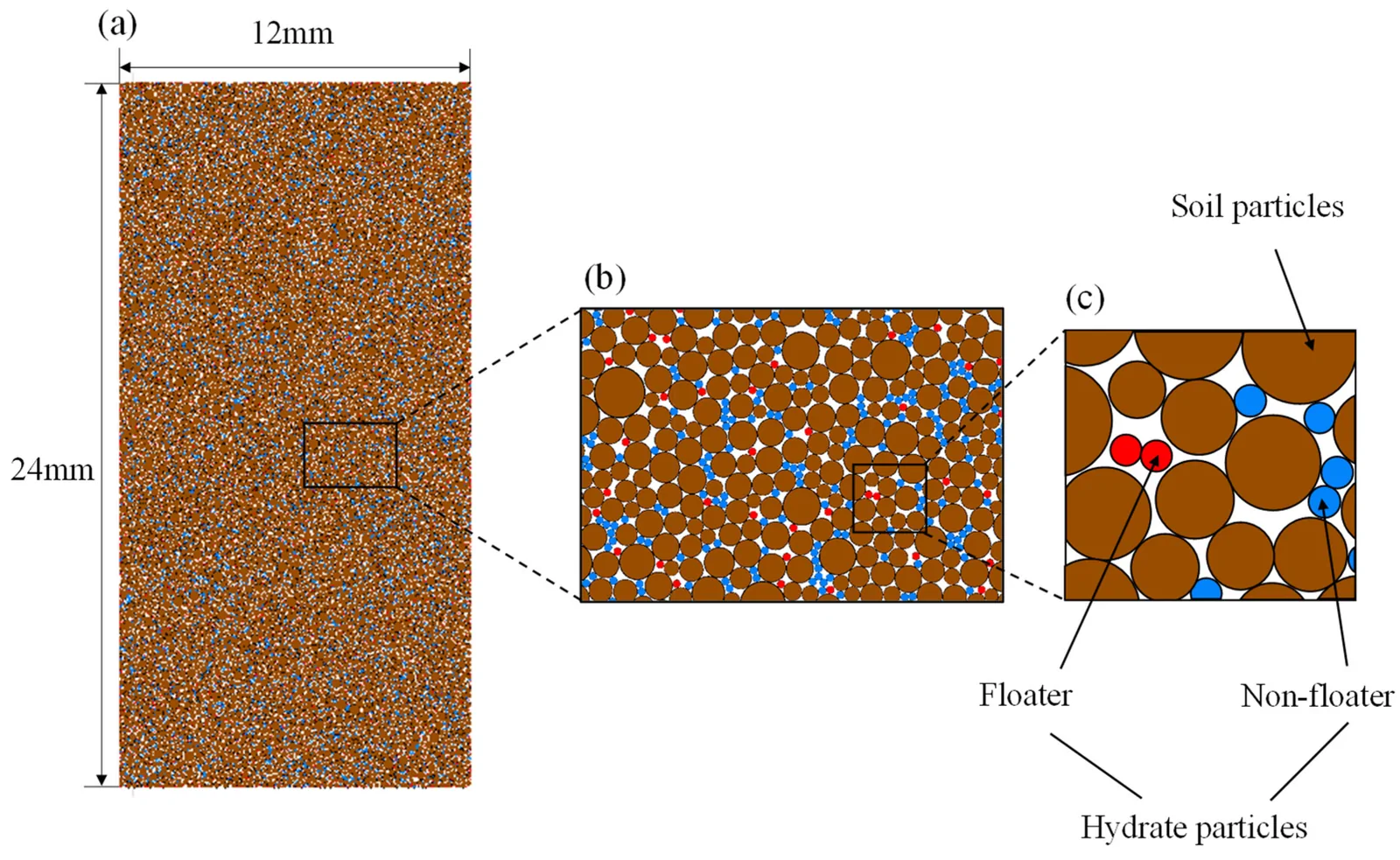
The initial sample of GHBS: (**a**) sample used in the simulation; (**b**) hydrate and soil particle arrangement; (**c**) partial enlargement.

**Figure 2:**
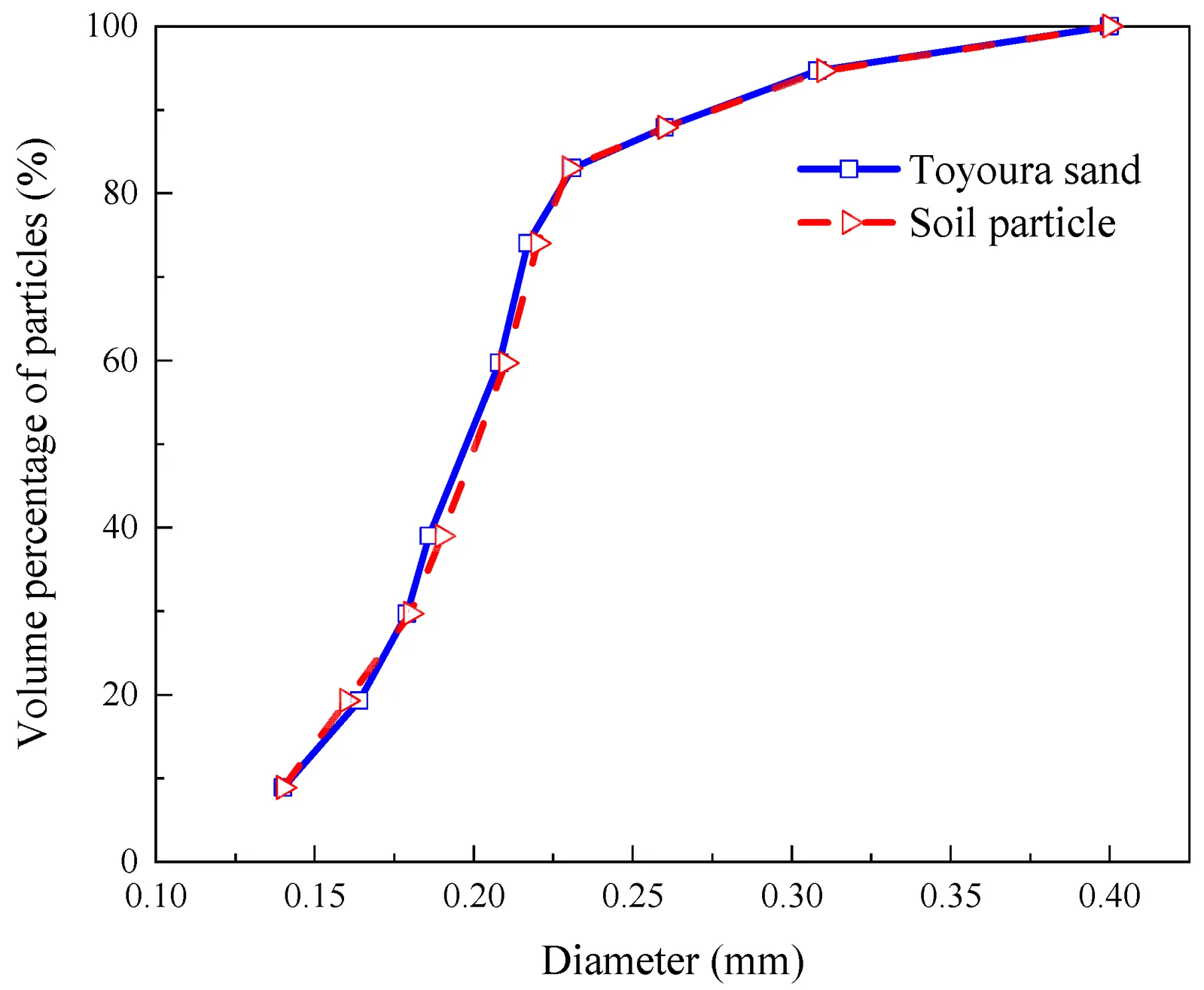
The curves of the particle size distribution of soil particles and Teymuraz sand.

**Figure 3:**
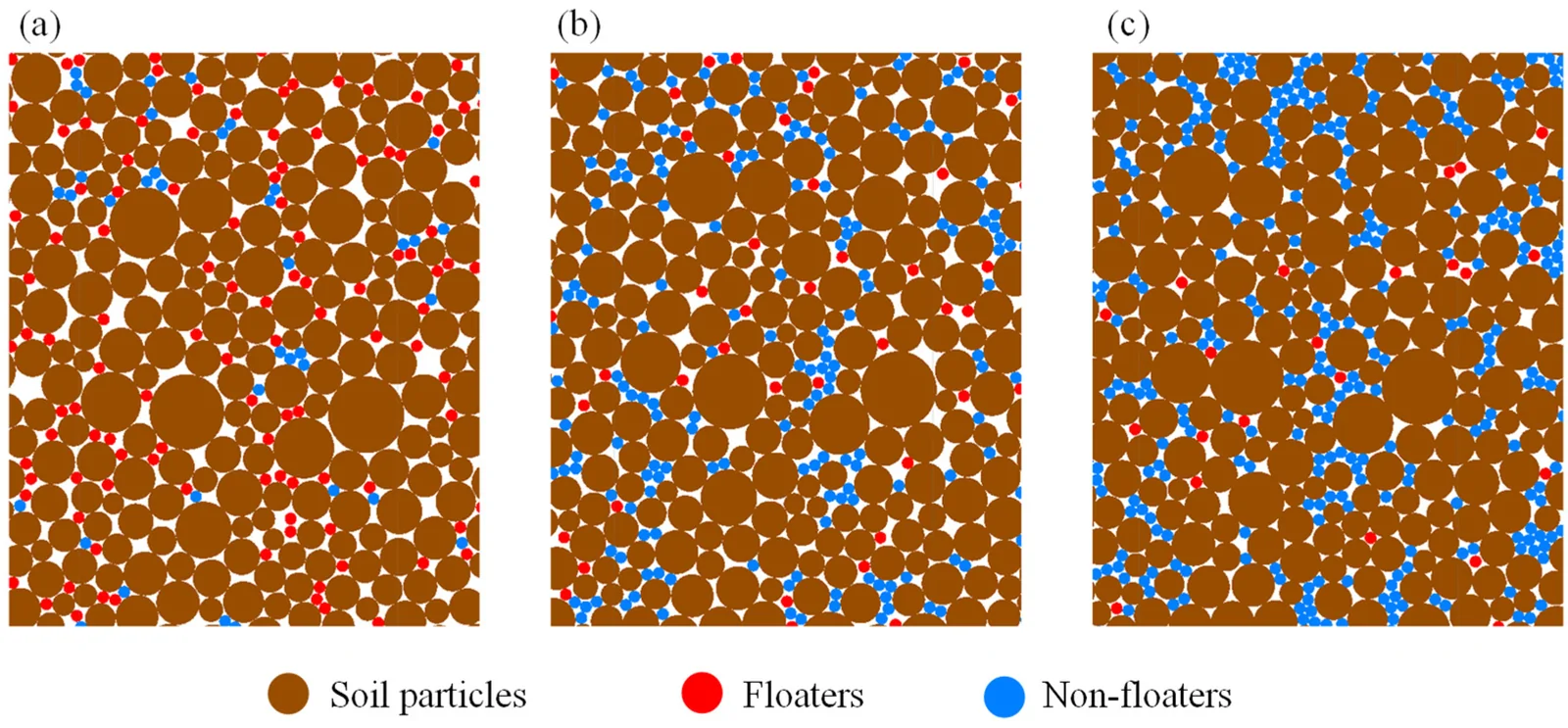
Local characteristics of samples with different hydrate saturation: (**a**) 26.0% hydrate saturation; (**b**) 40.0% hydrate saturation; (**c**) 55.0% hydrate saturation.

**Figure 4:**
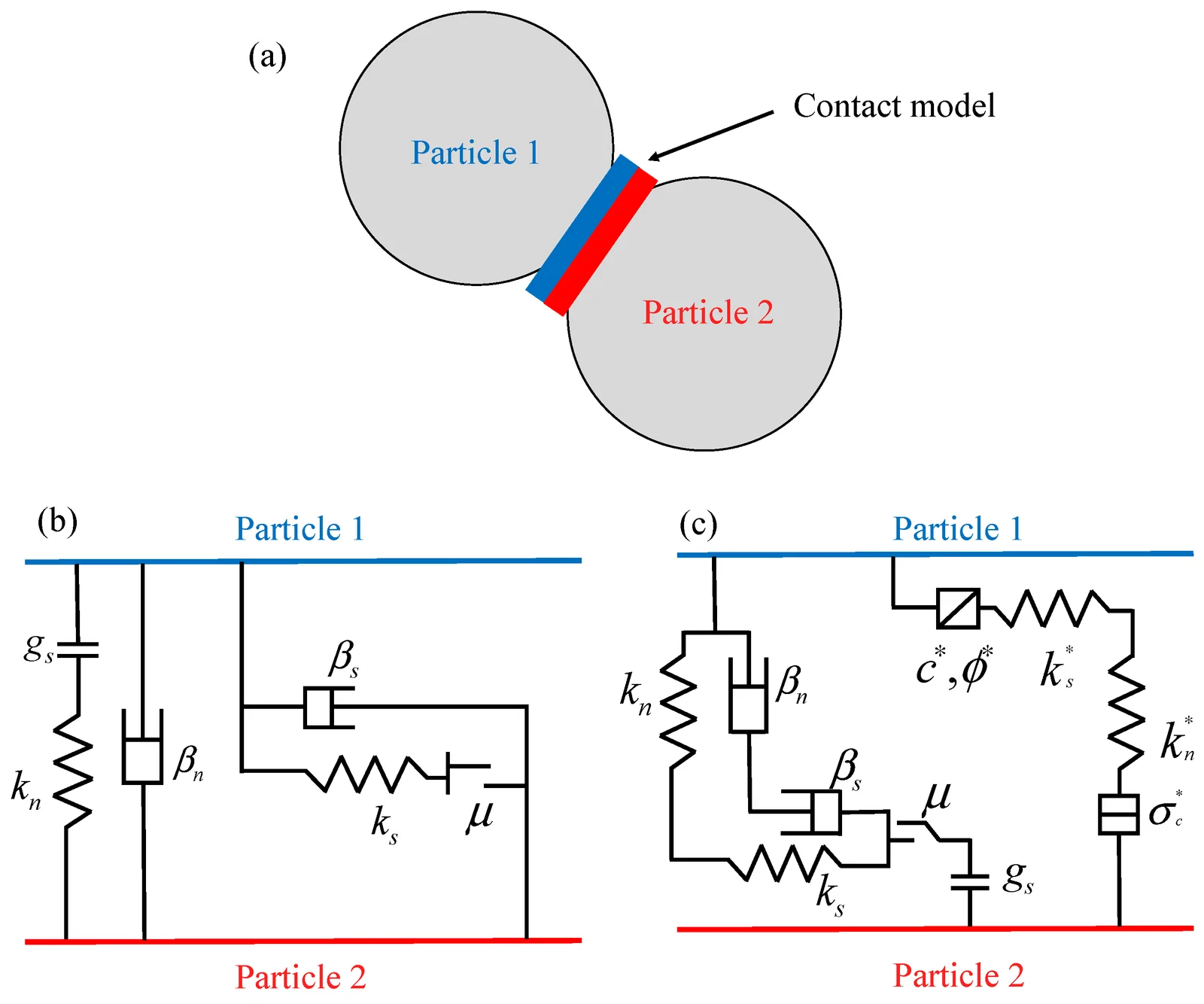
Mechanical components of the contact model: (**a**) location of the contact model; (**b**) linear model; (**c**) linear parallel bond model.

**Figure 5:**
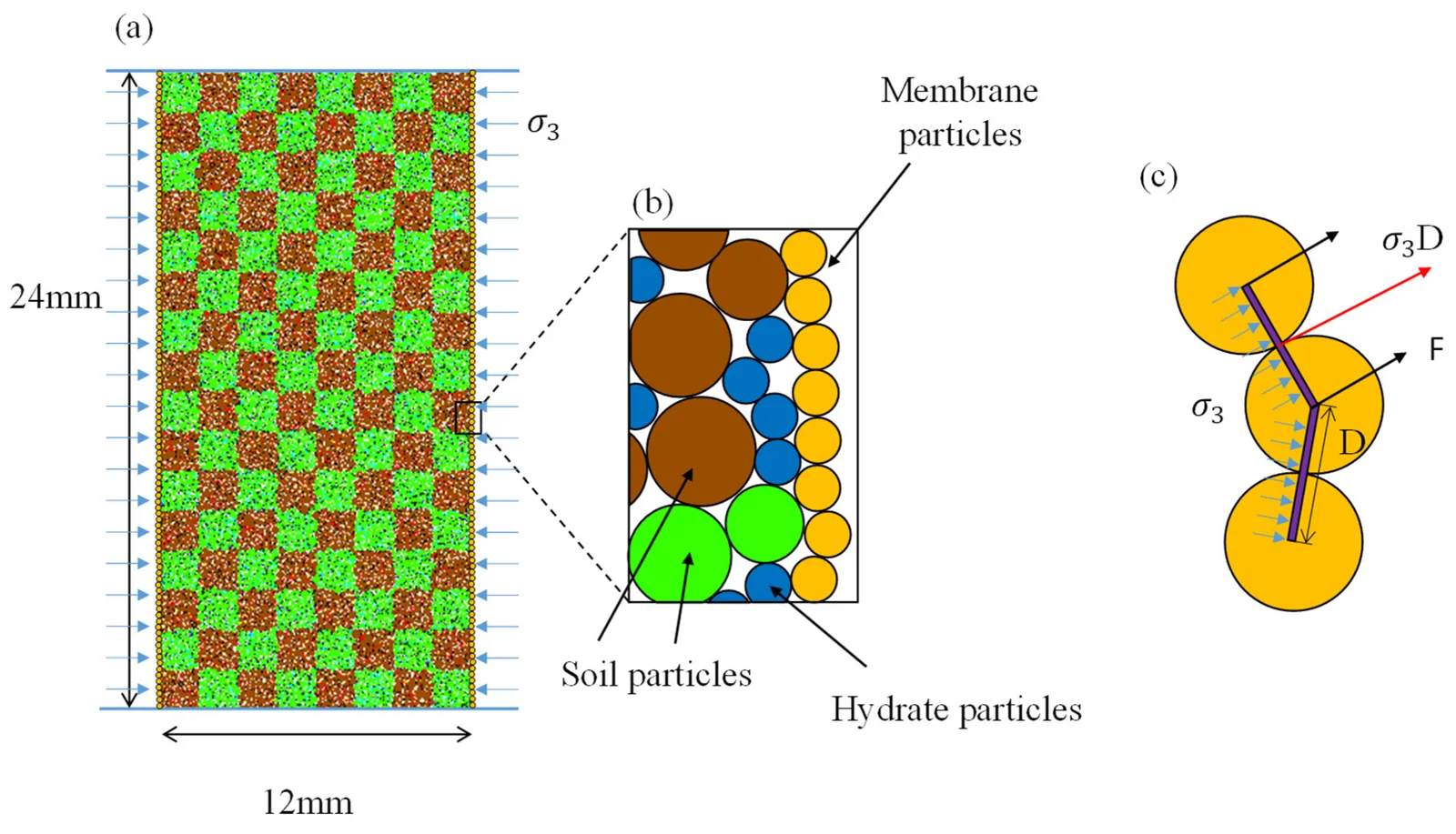
Layered stained sample: (**a**) sample under the membrane boundary condition; (**b**) membrane particles; (**c**) external force on membrane particles.

**Figure 6:**
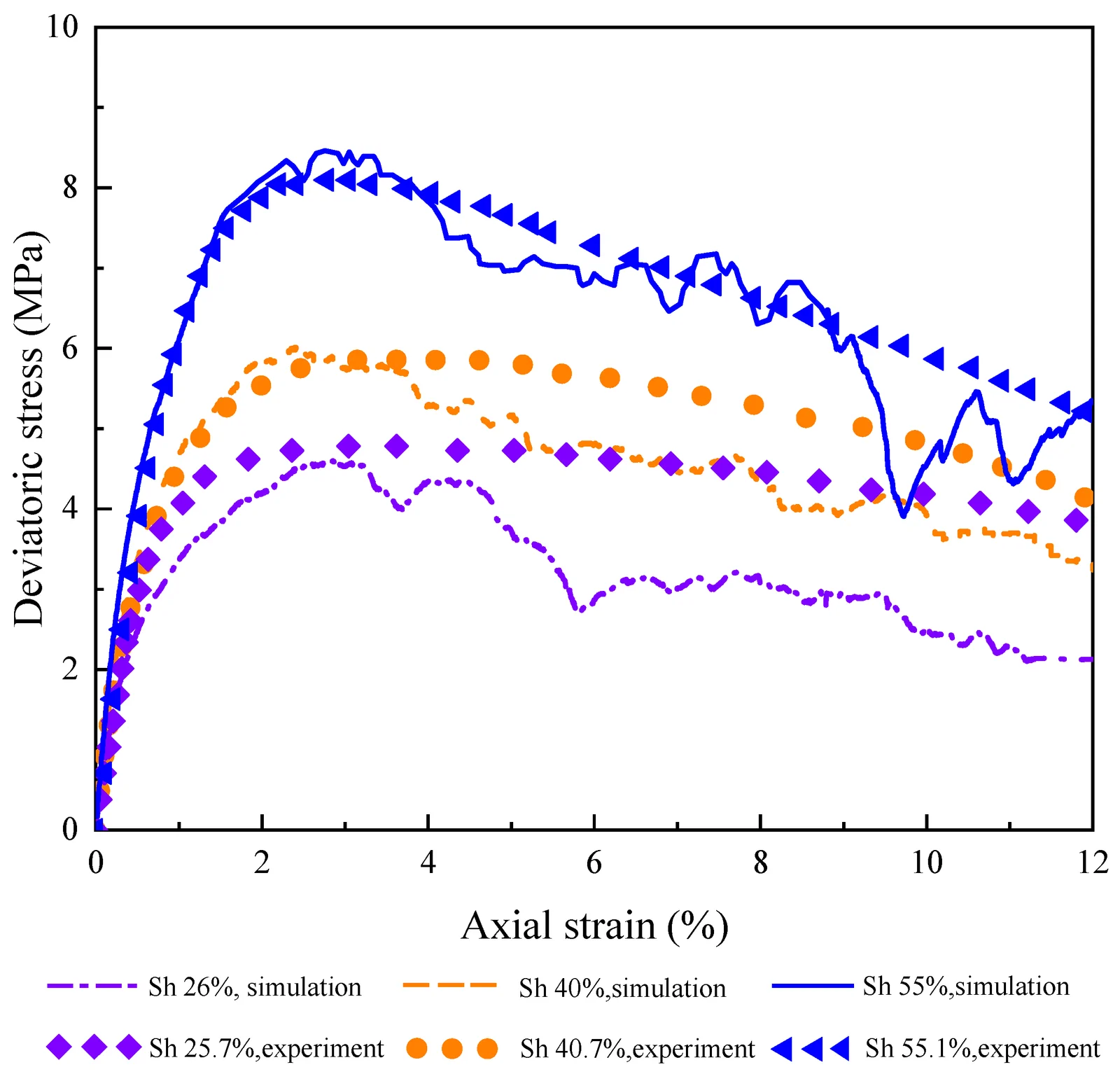
The curves of deviatoric stress versus axial strain of GHBS with different hydrate saturations.

**Figure 7:**
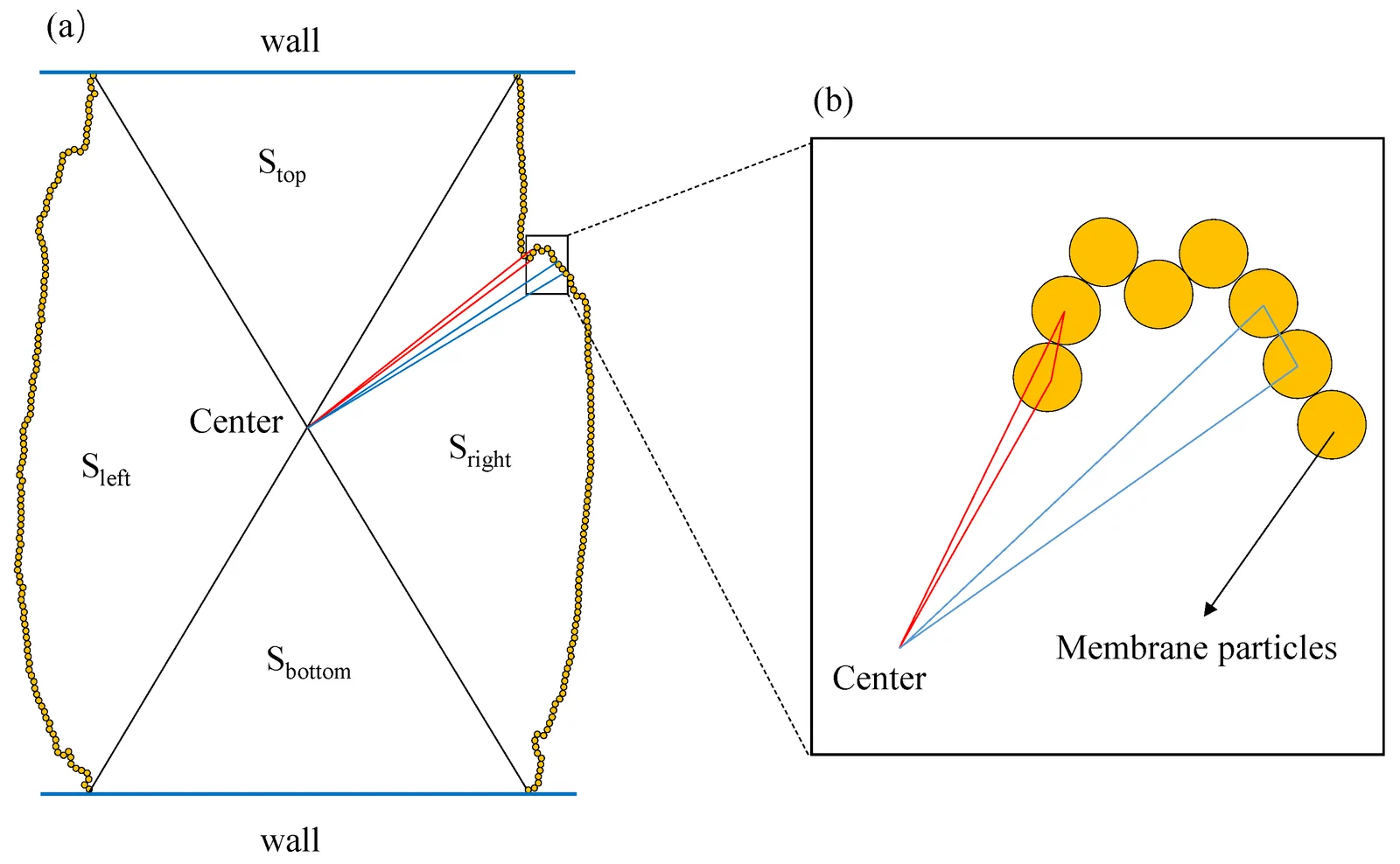
Schematic diagram of the triangle method for the deformed sample: (**a**) the geometric area division of the sample; (**b**) partial enlargement.

**Figure 8:**
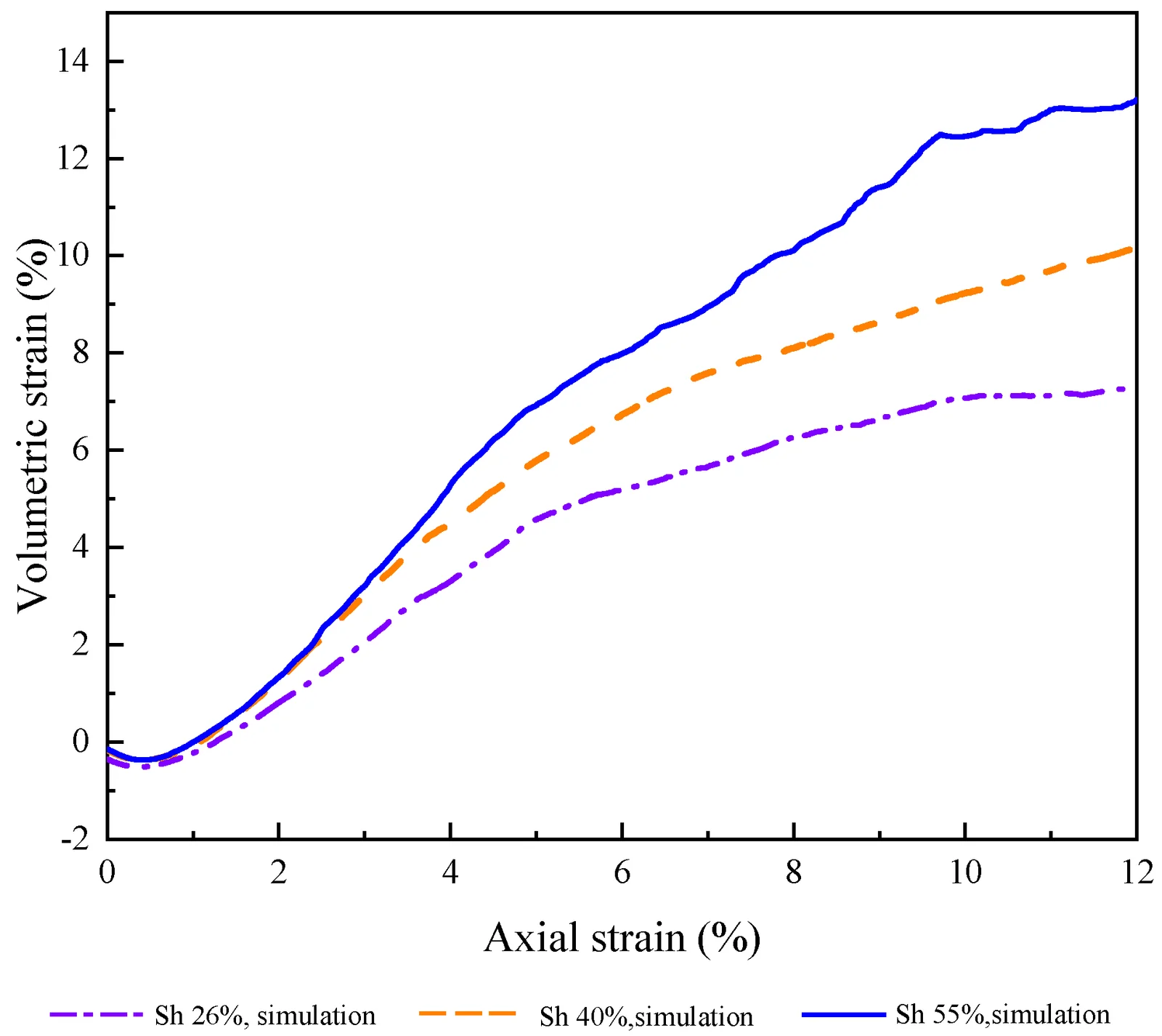
The curves of volumetric strain versus axial strain of GHBS with different hydrate saturations.

**Figure 9:**
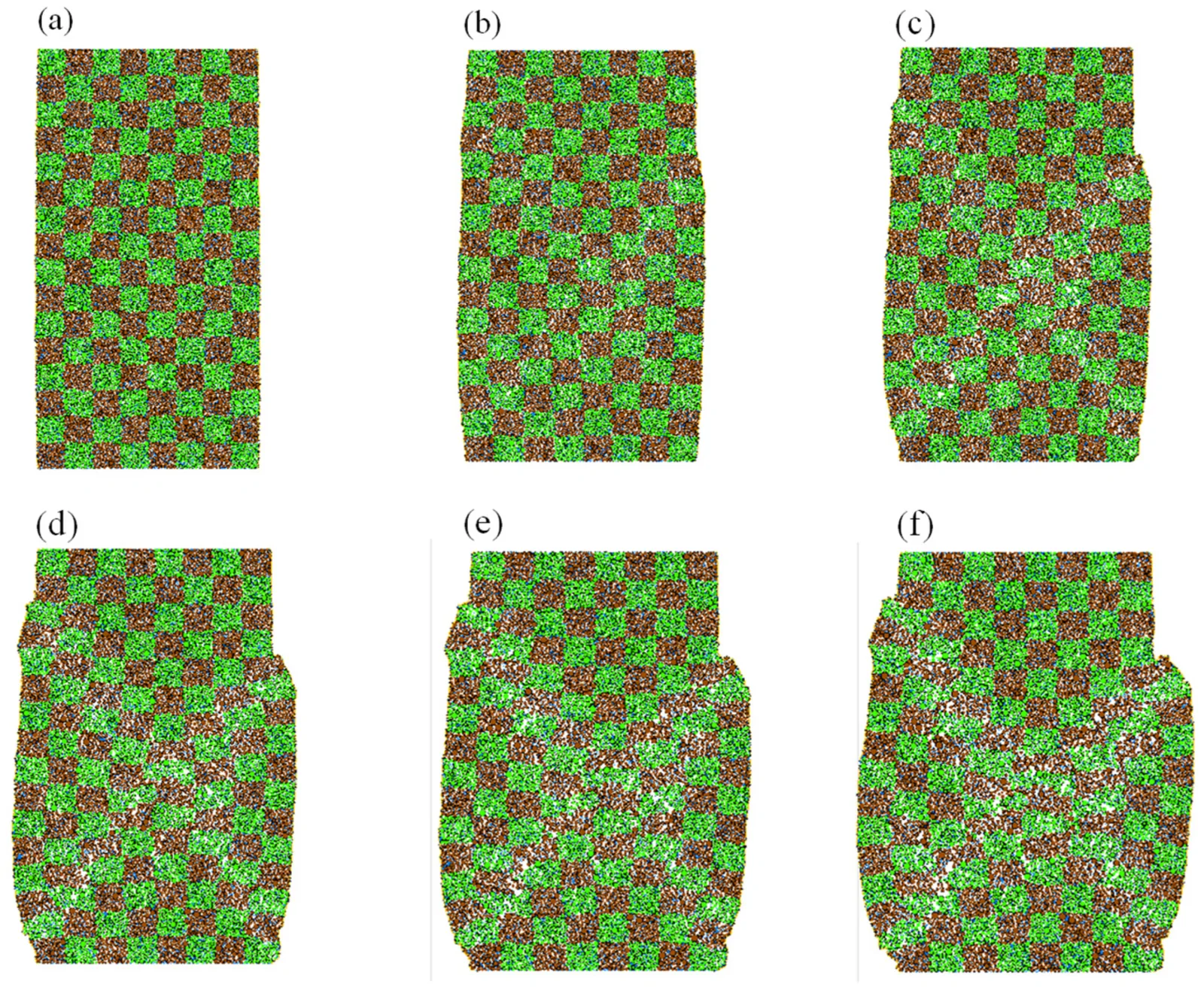
Evolution of the shear band: (**a**) axial strain 2%; (**b**) axial strain 4%; (**c**) axial strain 6%; (**d**) axial strain 8%; (**e**) axial strain 10%; (**f**) axial strain 12%.

**Figure 10:**
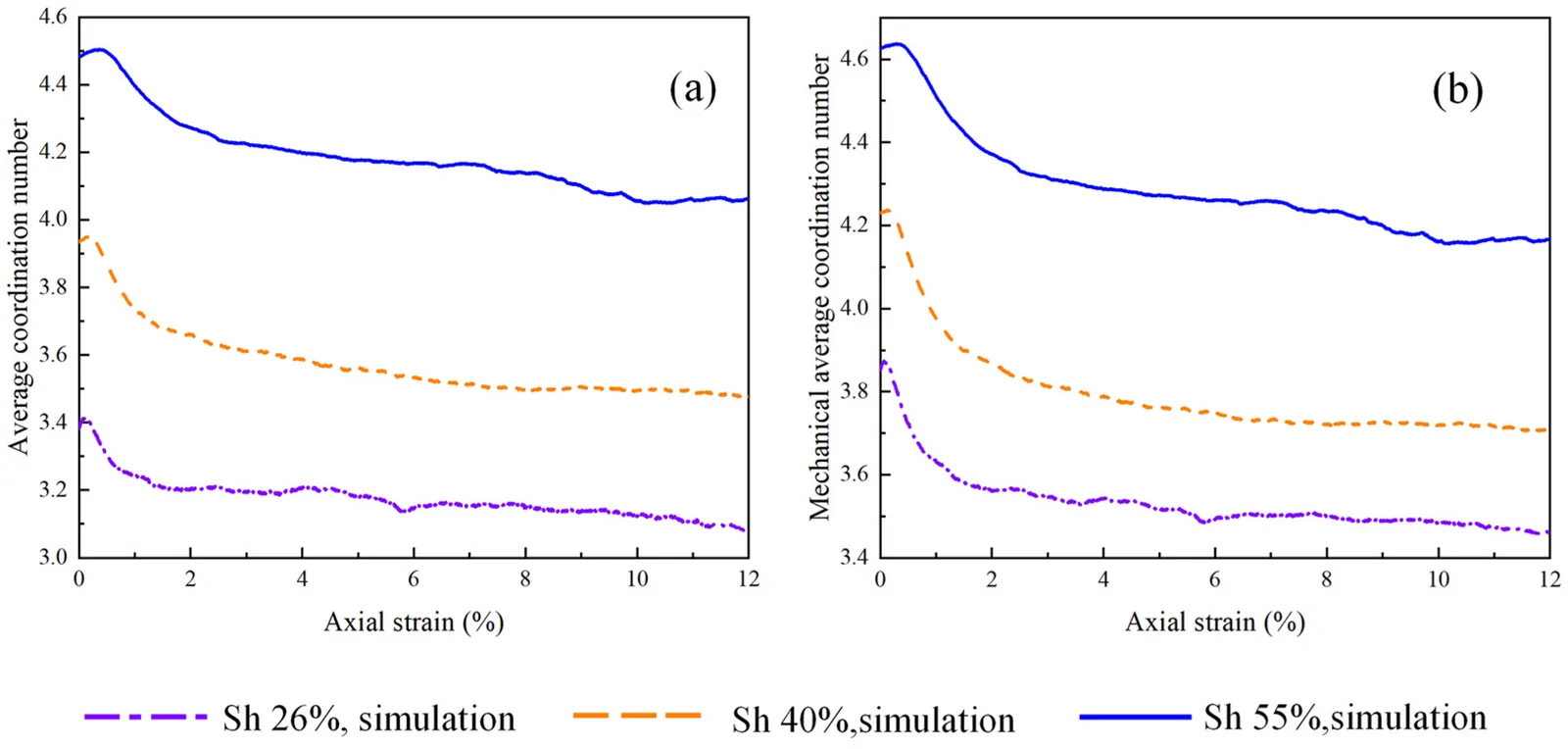
The curves of the evolution of coordination number of GHBS: (**a**) average coordination number; (**b**) mechanical average coordination number.

**Figure 11:**
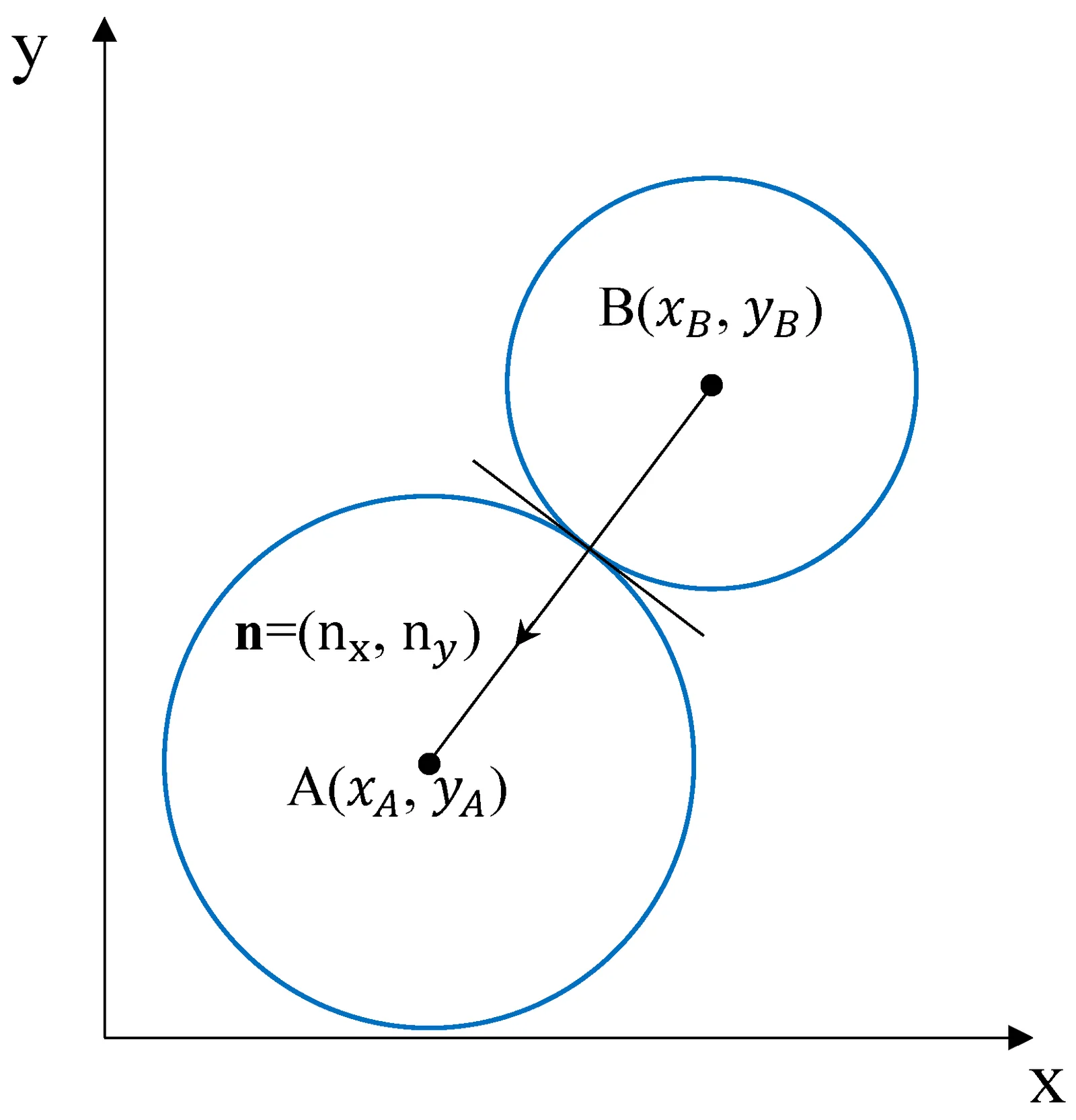
Schematic diagram of the unit contact normal between two particles.

**Figure 12:**
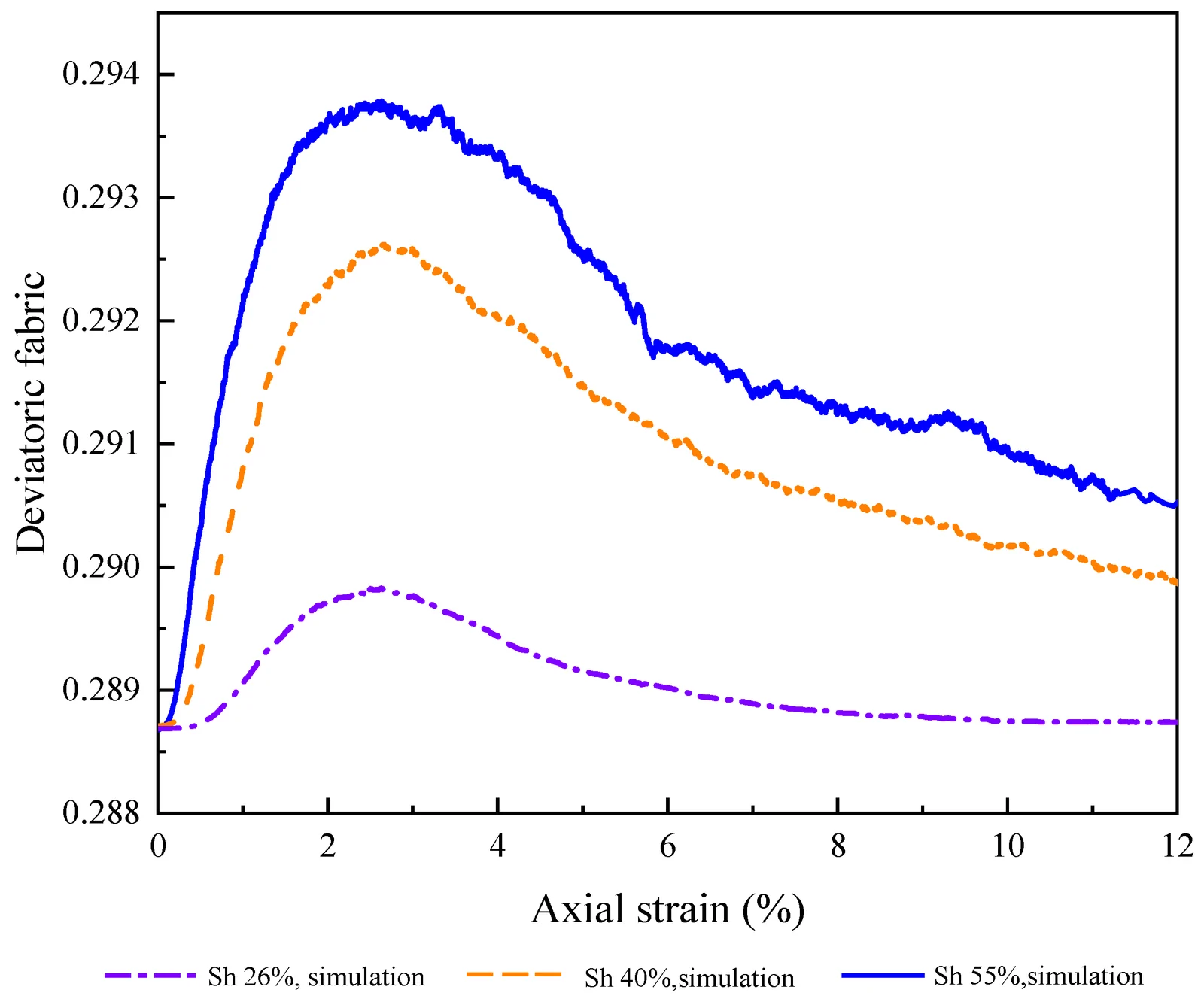
The curves of deviatoric fabric versus axial strain of the GHBS with different hydrate saturations.

**Figure 13:**
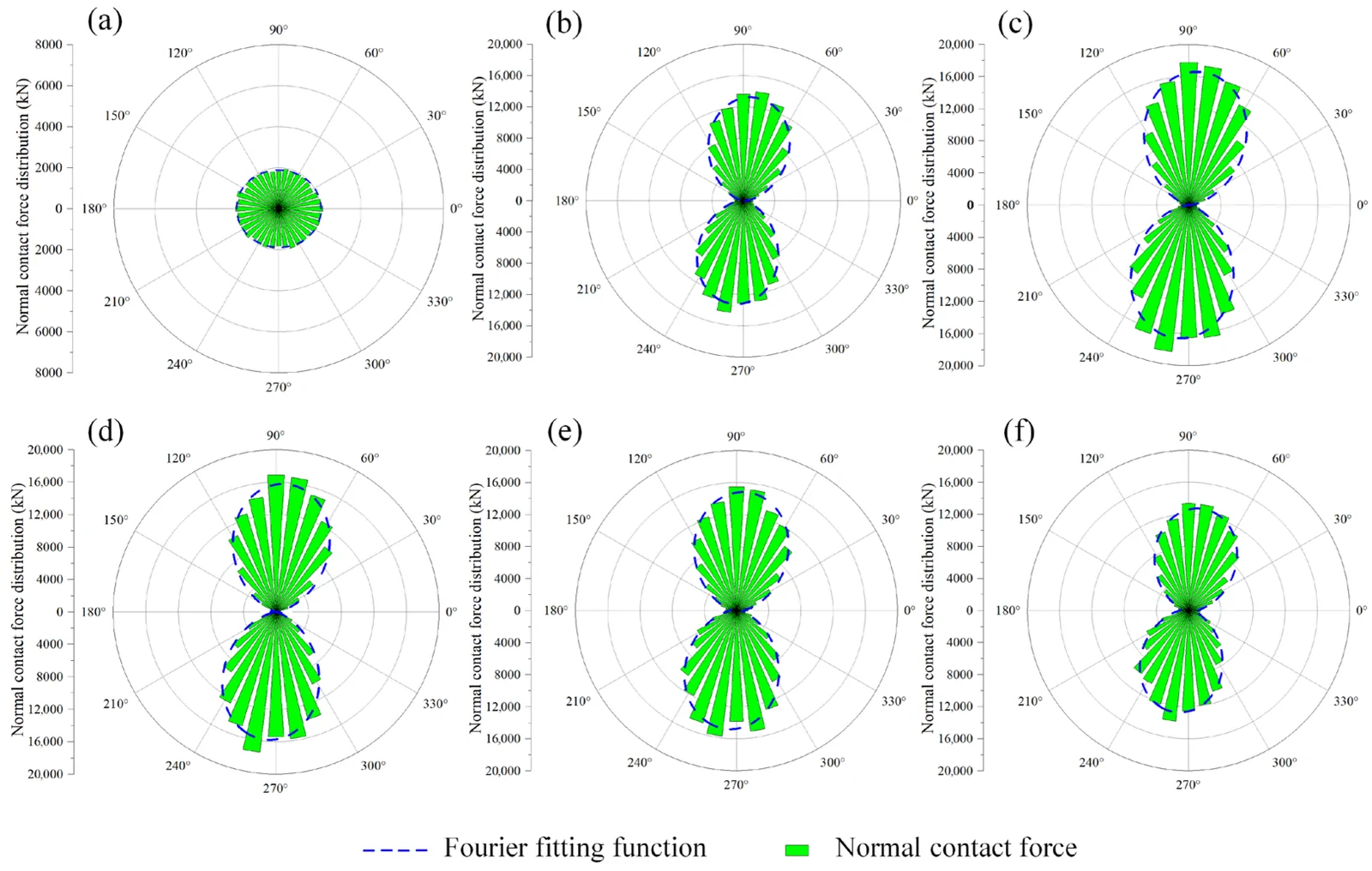
Distribution of normal contact force: (**a**) axial strain 0%; (**b**) axial strain 1%; (**c**) axial strain 2%; (**d**) axial strain 4%; (**e**) axial strain 8%; (**f**) axial strain 12%.

**Figure 14:**
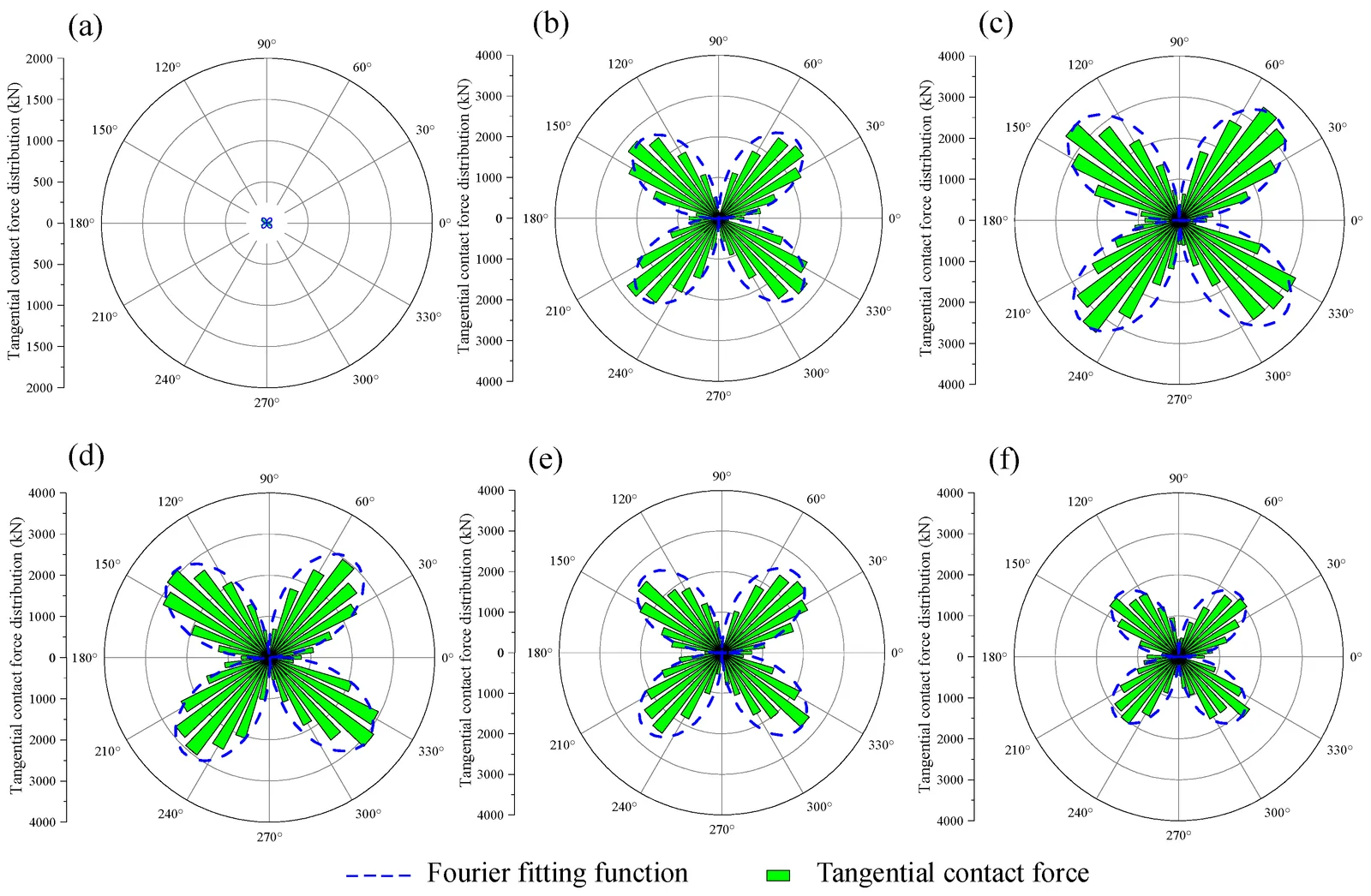
Distribution of tangential contact force: (**a**) axial strain 0%; (**b**) axial strain 1%; (**c**) axial strain 2%; (**d**) axial strain 4%; (**e**) axial strain 8%; (**f**) axial strain 12%.

**Figure 15:**
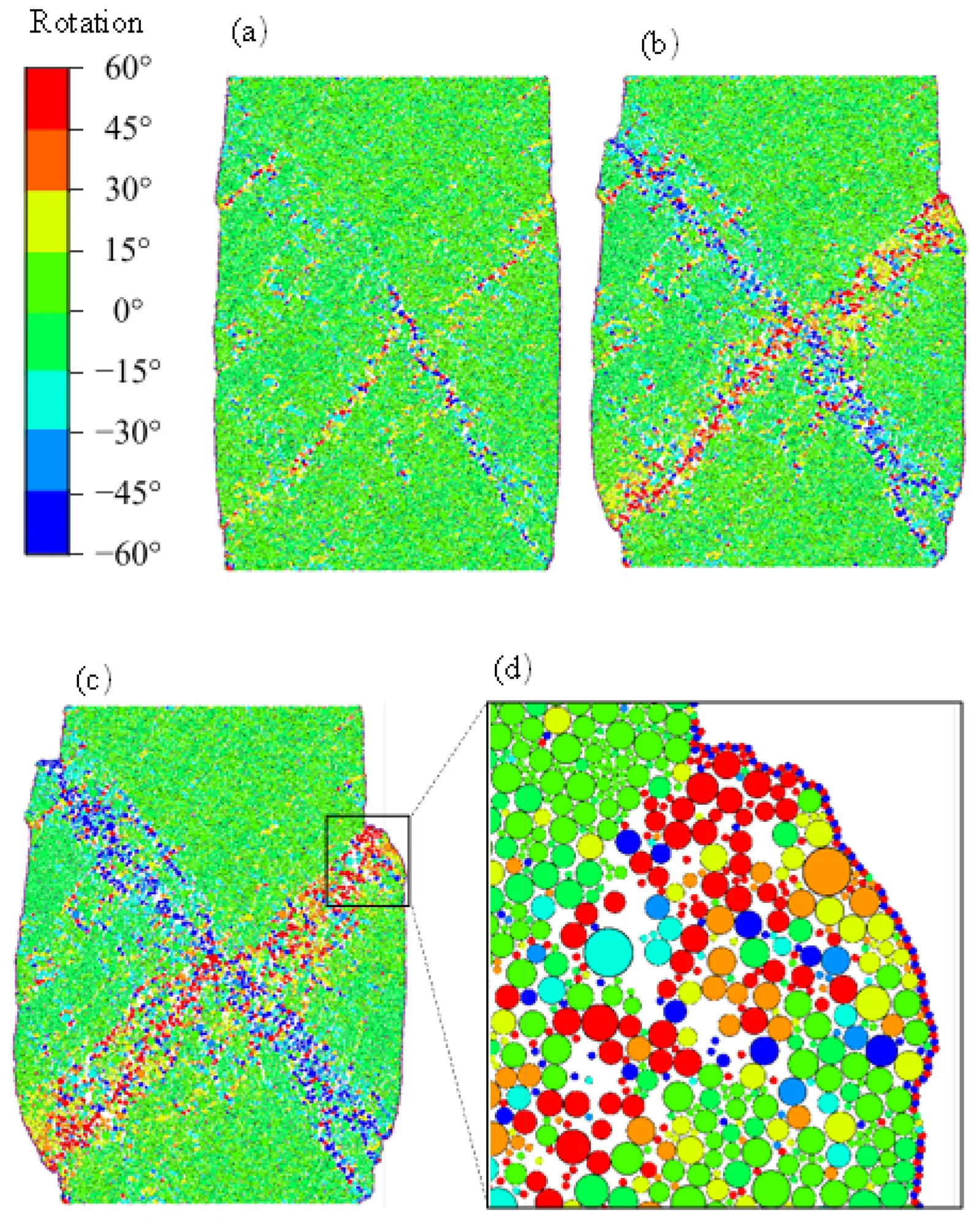
Distribution of particle rotation: (**a**) axial strain 4%; (**b**) axial strain 8%; (**c**) axial strain 12%; (**d**) partial enlargement.

**Table 1:** Parameters of contact models in the DEM simulations.

Contact Type	Contact Model Parameters	Values
Soil–soil contact	Effective modulus, *E*/MPa	1600.0
	Normal-to-shear stiffness ratio, *κ*	1.3
	Friction coefficient, *μ*	0.7
Hydrate–hydrate contact	Effective modulus, *E*/MPa	1200.0
	Normal-to-shear stiffness ratio, *κ*	1.3
	Friction coefficient, *μ*	0.5
	Parallel bond effective modulus, E*/MPa	25
	Parallel bond normal-to-shear stiffness ratio, κ*	1.3
	Parallel bond tensile strength, σ_c_*/MPa	50
	Parallel bond cohesion, c*/MPa	40
Soil–hydrate contact	Effective modulus, *E*/MPa	1200.0
	Normal-to-shear stiffness ratio, *κ*	1.3
	Friction coefficient, *μ*	0.5
	Parallel bond effective modulus, E*/MPa	25
	Parallel bond normal-to-shear stiffness ratio, κ*	1.3
	Parallel bond tensile strength, σ_c_*/MPa	50
	Parallel bond cohesion, c*/MPa	40
Wall	Effective modulus, *E*/MPa	1200.0
	Normal-to-shear stiffness ratio, *κ*	0.0
	Friction coefficient, *μ*	0.0

**Table 2:** Fitting parameters of distribution of contact force.

Axial Strain	*f*_0_/N	Normal Contact Force		Tangential Contact Force	
		*a_n_*	*θ_n_*/°	*a_t_*	*θ_t_*
0%	1.96 × 10^6^	−0.043	86.0	0.00010	0
1%	6.72 × 10^6^	0.97	85.0	0.40	1
2%	8.14 × 10^6^	1.04	84.0	0.43	3
4%	8.04 × 10^6^	1.02	86.0	0.39	4
8%	7.77 × 10^6^	0.90	86.0	0.34	2
12%	6.73 × 10^6^	0.87	83.0	0.31	3

## Data Availability

The original contributions presented in this study are included in the article. Further inquiries can be directed to the corresponding author.
